# Absolving COVID-19 Vaccination of Autoimmune Bullous Disease Onset

**DOI:** 10.3389/fimmu.2022.834316

**Published:** 2022-02-18

**Authors:** Roberto Russo, Giulia Gasparini, Emanuele Cozzani, Federica D’Agostino, Aurora Parodi

**Affiliations:** ^1^ Dipartimento di Scienze della Salute (DISSAL), Section of Dermatology, University of Genoa, Genoa, Italy; ^2^ Department of Dermatology, San Martino Polyclinic Hospital, Genoa, Italy

**Keywords:** COVID-19, vaccines, autoimmune bullous diseases, bullous pemphigoid, pemphigus vulgaris

## Introduction

Even before the outbreak of coronavirus disease 2019 (COVID-19), vaccines have been the most important preventive measure against infectious diseases. Vaccines have led to the eradication of some infectious diseases and have reduced the mortality and morbidity of many others. However, concerns about their safety have led to hesitancy toward vaccination. Despite their undeniable advantages, vaccinations have also been accused of having a potential role in inducing autoimmune diseases (1, 2).

Some cases of arthritis, vasculitis, and central or peripheral nervous system disorders have been reported following vaccination (3). As concerns the COVID-19 vaccine, the matter is further complicated by the nucleic acid formulation of the vaccine and its accelerated development imposed by the emergency situation of the pandemic (4). On one hand, patients with autoimmune diseases are more likely to develop severe forms of COVID-19 rather than adverse reactions to the COVID-19 vaccine (5); on the other hand, several reports have been linking COVID-19 vaccination with the onset of a variety of autoimmune disorders (6–8). As for the dermatological adverse reactions, among others (9), recent reports suggest an association between COVID-19 vaccination and the onset of autoimmune bullous diseases (AIBDs) (10–13). Should we really blame the vaccines?

## An Example

As a perfect example of what we mean, we would like to cite the case of a 75-year-old male with type-II diabetes mellitus treated with gliptins, who developed bullous pemphigoid (BP) 48 h after receiving the first dose of the Comirnaty Pfizer-BioNTech vaccine. Physical examination revealed widespread tense blisters on erythematous skin. Histological examination and direct immunofluorescence findings confirmed the diagnosis of BP. Discontinuation of gliptins resulted in prompt clinical improvement, and the cutaneous lesions were managed by topical steroids alone.

## Discussion

Vaccine-associated autoimmunity is a well-known phenomenon, which can be attributed to either the cross-reactivity between antigens or the effect of adjuvants (4). Reports on the onset of autoimmune diseases, including AIBDs, in healthy patients who received COVID-19 vaccines have been recently published. Pérez-López et al. (10) reported the case of a 78-year-old woman with diabetes mellitus and Alzheimer’s disease, treated with insulin and memantine, who developed BP 3 days after receiving the first dose of the Comirnaty Pfizer-BioNTech vaccine. Notably, she experienced a subsequent reactivation after receiving the second dose (10). A 77-year-old patient, with no significant clinical history, consulted for skin blisters that appeared 24 h after the first injection of the AstraZeneca COVID-19 vaccine; immunofluorescence findings confirmed the diagnosis of BP (11). Solimani et al. (12) described the case of a 40‐year‐old Asian woman, without any history of skin disease and otherwise healthy, who developed pemphigus vulgaris (PV) 5 days after COVID‐19 vaccination with the Comirnaty Pfizer-BioNTech vaccine; also, the lesions worsened and spread to the upper body 3 days after the second dose. Damiani et al. (13) highlighted one more issue, by arguing that COVID‐19 vaccines may trigger PV and BP flares in patients already affected by these diseases, during a period of remission. Namely, 5 patients (2 PV, 3 BP) were reported to have experienced a flare after undergoing COVID-19 vaccination (13).

Our patient developed BP shortly after receiving the first dose of Comirnaty Pfizer-BioNTech vaccine. On the other hand, the association between gliptins and BP is well-known (14). Therefore, BP might be considered as an adverse effect of the antidiabetic treatment. Moreover, discontinuing gliptins resulted in quick clinical improvement, giving strength to the hypothesis of a drug adverse event. However, one may argue that the onset only 48 h after the immunization suggests a role played by the vaccine, as the patient had been taking gliptins for 1 year without any skin reactions. In our opinion, our case suggests the role of COVID-19 vaccine as a possible trigger, but not as an independent cause, of immune reaction in the setting of a gliptin-related BP.

Also, when considering COVID-19 vaccination as a possible cause of AIBDs, one would expect an increase of their incidence in 2021 compared to the previous years. Oppositely, in our department which is a regional referral center for AIBDs, we observed a stable, or rather an inverse, trend, as 33 new cases of AIBDs (of which 25 were BP) were diagnosed in 2021 compared to 41 cases (31 BP) in 2020 and 47 cases (26 BP) in 2019. This appears to give strength to our hypothesis that COVID-19 vaccines probably are not independent causes of AIBDs.

Of course, well-conducted epidemiological studies are needed to clarify whether an actual relationship between COVID-19 vaccines and AIBDs really exists. However, perhaps some of the AIBDs reported to be vaccine-induced actually are not. In fact, as in our case, sometimes there are underlying factors that make the patient prone to develop AIBDs, and vaccination only accidentally occurs shortly before the AIBD onset, or at most, it acts as a triggering event. Unfortunately, not all these underlying factors of BP and PV are known, so AIBDs may be incorrectly considered as vaccine-induced. This is of crucial importance, as it may result in improper contraindication to further doses. Our patient was advised to get the second dose of the vaccine, but he refused.

In conclusion, benefits of vaccination against COVID-19 outweigh risks in terms of developing AIBDs; therefore, dermatologists should advise their patients to get vaccinated.

## Author Contributions

All authors contributed equally in all phases of work. All authors contributed to the article and approved the submitted version.

## Conflict of Interest

The authors declare that the research was conducted in the absence of any commercial or financial relationships that could be construed as a potential conflict of interest.

## Publisher’s Note

All claims expressed in this article are solely those of the authors and do not necessarily represent those of their affiliated organizations, or those of the publisher, the editors and the reviewers. Any product that may be evaluated in this article, or claim that may be made by its manufacturer, is not guaranteed or endorsed by the publisher.

## References

[1] Principi N Esposito S. Do Vaccines Have a Role as a Cause of Autoimmune Neurological Syndromes? Front Public Health (2020) 8:361. doi: 10.3389/fpubh.2020.00361 32850592 PMC7399175

[2] Guimarães LE Baker B Perricone C Shoenfeld Y. Vaccines, Adjuvants and Autoimmunity. Pharmacol Res (2015) 100:190–209. doi: 10.1016/j.phrs.2015.08.003 26275795 PMC7129276

[3] Toussirot É Bereau M. Vaccination and Induction of Autoimmune Diseases. Inflamm Allergy Drug Targets (2015) 14:94–8. doi: 10.2174/1871528114666160105113046 26728772

[4] Talotta R. Do COVID-19 RNA-Based Vaccines Put at Risk of Immune-Mediated Diseases? In Reply to “Potential Antigenic Cross-Reactivity Between SARS-CoV-2 and Human Tissue With a Possible Link to an Increase in Autoimmune Diseases”. Clin Immunol (2021) 224:108665. doi: 10.1016/j.clim.2021.108665 33429060 PMC7833091

[5] Cozzani E Gasparini G Sticchi L Russo R Icardi G Parodi A. Considerations on SARS-Cov-2 Vaccines in Patients With Autoimmune Blistering Diseases. Eur J Dermatol (2021) 31:415–7. doi: 10.1684/ejd.2021.4043 PMC8354837 34309530

[6] Okuno S Hashimoto K Shimizu R Takagi E Kajiguchi T. Development of Autoimmune Hemolytic Anemia After BNT162b2 mRNA COVID-19 Vaccination. Rinsho Ketsueki (2021) 62:1510–4. doi: 10.11406/rinketsu.62.1510 34732625

[7] Tan CK Wong YJ Wang LM Ang TL Kumar R. Autoimmune Hepatitis Following COVID-19 Vaccination: True Causality or Mere Association? J Hepatol (2021) 75:1250–2. doi: 10.1016/j.jhep.2021.06.009 PMC8404983 34153398

[8] Tarawneh O Tarawneh H. Immune Thrombocytopenia in a 22-Year-Old Post Covid-19 Vaccine. Am J Hematol (2021) 96:133–4. doi: 10.1002/ajh.26106 PMC8014773 33476455

[9] Burlando M Russo R Cozzani E Parodi A. COVID-19 "Second Wave" and Vaccines: The Dermatologists’ Perspective. Int J Dermatol (2021) 60:889–90. doi: 10.1111/ijd.15547 PMC8251220 33763883

[10] Pérez-López I Moyano-Bueno D Ruiz-Villaverde R. Bullous Pemphigoid and COVID-19 Vaccine. Med Clin (Barc) (2021) 157:333–4. doi: 10.1016/j.medcle.2021.05.004 PMC8529265 34697598

[11] Agharbi FZ Eljazouly M Basri G Faik M Benkirane A Albouzidi A. Bullous Pemphigoid Induced by the AstraZeneca COVID-19 Vaccine. Ann Dermatol Venereol (2021):S0151 9638(21)00091-0. doi: 10.1016/j.annder.2021.07.008 PMC8481089 34686374

[12] Solimani F Mansour Y Didona D Dilling A Ghoreschi K Meier K. Development of Severe Pemphigus Vulgaris Following SARS-CoV-2 Vaccination With Bnt162b2. J Eur Acad Dermatol Venereol (2021) 35:649–51. doi: 10.1111/jdv.17480 PMC8447452 34169588

[13] Damiani G Pacifico A Pelloni F Iorizzo M. The First Dose of COVID-19 Vaccine may Trigger Pemphigus and Bullous Pemphigoid Flares: Is the Second Dose Therefore Contraindicated? J Eur Acad Dermatol Venereol (2021) 35:645–7. doi: 10.1111/jdv.17472 PMC8447358 34169578

[14] Verheyden MJ Bilgic A Murrell DF. A Systematic Review of Drug-Induced Pemphigoid. Acta Derm Venereol (2020) 100:adv00224.v. doi: 10.2340/00015555-3457 32176310 PMC9207627

